# Pathogenic Loss-of-Function Germline *TERT* Mutations in Patients With Solid Tumors

**DOI:** 10.1200/PO.19.00230

**Published:** 2019-10-23

**Authors:** Michael F. Walsh, Rosalba Sacca, Temima Wildman, Kimberly Amoroso, Jennifer Kennedy, Liying Zhang, Ozge Birsoy, Diana Mandelker, Zoe Steinsnyder, Alicia Latham, Maria I. Carlo, Karen Cadoo, Yelena Kemel, Mark Robson, Zsofia K. Stadler, Kenneth Offit

**Affiliations:** ^1^ Memorial Sloan Kettering Cancer Center, New York, NY; ^2^ Weill Cornell Medical College, New York, NY

## INTRODUCTION

Dyskeratosis congenita, the original telomere syndrome, was clinically described more than 100 years ago on the basis of individuals who presented with a distinct rash, abnormal nails, and whitening of the tongue.^1^ From this rare syndrome, our clinical and molecular understanding of telomere syndromes has evolved and has now expanded to include aplastic anemia, myelodysplastic syndrome, and pulmonary fibrosis.^2-5^ The identification of patients with telomere syndromes is of significant clinical importance because these patients are exquisitely sensitive to alkylating chemotherapeutic agents and ionizing radiation.^6-8^

Precision medicine efforts that deploy tumor-normal sequencing have used various molecular assays and platforms to interrogate and annotate the cancer census genes.^9,10^ *TERT,* a gene that encodes a key protein involved in telomere maintenance, has been analyzed predominantly to identify genomic alterations that occur in tumors.^11,12^ Although constitutional telomere syndromes are recognized, they are rarely considered in the oncologist’s differential diagnosis unless a diagnosis of a telomere syndrome occurred before the patient’s cancer diagnosis.^13^

From January 2016 to May 2019, unselected patients with advanced solid tumors were presented with the option to participate and consent to a Memorial Sloan Kettering Cancer Center institutional review board–approved protocol (#12-245; ClinicalTrials.gov identifier: NCT01775072) of tumor and germline DNA sequencing. Patients viewed a standard pretest educational video on germline genetic testing. All patients with pathogenic or likely pathogenic variants were offered genetic counseling. Variants of uncertain significance were not reported. Electronic medical records were reviewed for demographic and clinical variables, including family history.

Here, we describe the frequency of individuals with germline *TERT* mutations and their associated clinical characteristics in the first 11,096 individuals who underwent agnostic germline molecular testing, and for four (57.1%) of seven individuals in this group with germline *TERT* mutations, telomere length assessment was possible (Table 1).

**TABLE 1.**
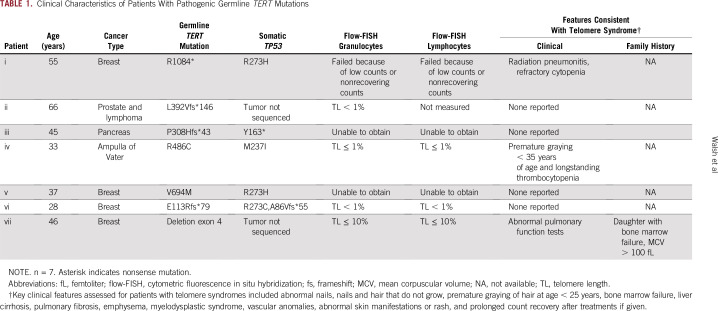
Clinical Characteristics of Patients With Pathogenic Germline *TERT* Mutations

## MOLECULAR METHODS

### Sequencing, Variant Calling, and Results Reporting

Memorial Sloan Kettering–Integrated Mutation Profiling of Actionable Cancer Targets (MSK-IMPACT), a 468-gene targeted capture panel, was used for tumor sequencing, while germline analysis included initially a 76-gene and then an 88-gene hereditary predisposition panel^9,14,15^ (Data Supplement). All variants with less than 1% population frequency in the Exome Aggregation Consortium (ExAC) database were interpreted. A clinical molecular geneticist or molecular pathologist interpreted variants per American College of Medical Genetics criteria.^16^ Mutations were classified as high (relative risk [RR], > 4), moderate (RR, 2 to 4), or low (RR, < 2) penetrance or as recessive.

### Comparison of Germline Data to Public Databases

To assess associations of specific variants and tumor phenotype, population allele frequencies (AFs) in cases were compared with AFs in noncancer cases obtained from the ExAC^17^ public database minus cases obtained from The Cancer Genome Atlas.^14^ Comparisons of AFs in Ashkenazi Jewish cases were restricted to Ashkenazi Jewish individuals in the genome aggregation database release 2.01.^18^ AFs were compared by Fisher’s exact test in R version 3.3.2 using RStudio version 1.0.136 (RStudio, Boston, MA) to compute the odds ratios, CIs, and *P* values. Clinical variables in subsets defined by mutation status were compared by analysis of variance using GraphPad Prism version 7.01 software (GraphPad Software, San Diego, CA).^19-21^

### *TERT* Sequencing

The average coverage of *TERT* was a minimum of more than 150× coverage to comply with quality control standards. The Memorial Sloan Kettering Cancer Center germline pipeline does not call *TERT* promoter variants as does the somatic pipeline, but it does call exons with ± 20 base pairs.^9^

### Telomere Lengths

To assess telomere length, peripheral blood lymphocytes and granulocytes were sent to RepeatDx (North Vancouver, British Columbia, Canada) and measured through cytometric fluorescence in situ hybridization.^22,23^

## CASE SERIES

Of 11,096 individuals who underwent MSK-IMPACT testing from January 2016 to May 2019, seven were found to harbor a germline pathogenic variant in *TERT*. Cancer types in our cohort included four female patients with breast cancer (ages at diagnosis, 28, 37, 46, and 58 years), one male patient with gall bladder adenocarcinoma (age 33 years), one male patient with pancreatic ampullary adenocarcinoma (age 45 years), and one male patient with two malignancies: lymphoma at age 58 years and prostate cancer at age 66 years. In the six patients with a solid tumor as their first malignancy, all were younger than the median age of diagnosis in the general population for their type of cancer^24-27^ (Data Supplement).

Clinical histories of three (42.8%) of the seven patients were suggestive of telomere syndromes. Individual i, diagnosed with breast cancer, endured therapy-induced radiation pneumonitis and refractory thrombocytopenia; individual iv, diagnosed with ampulla of Vater cancer, experienced premature graying and thrombocytopenia that preceded a cancer diagnosis at age 19 years; and individual vii revealed a history of bone marrow failure in a family member with a segregating *TERT* mutation and showed abnormal pulmonary function testing (Table 1).

**FIG 1.**
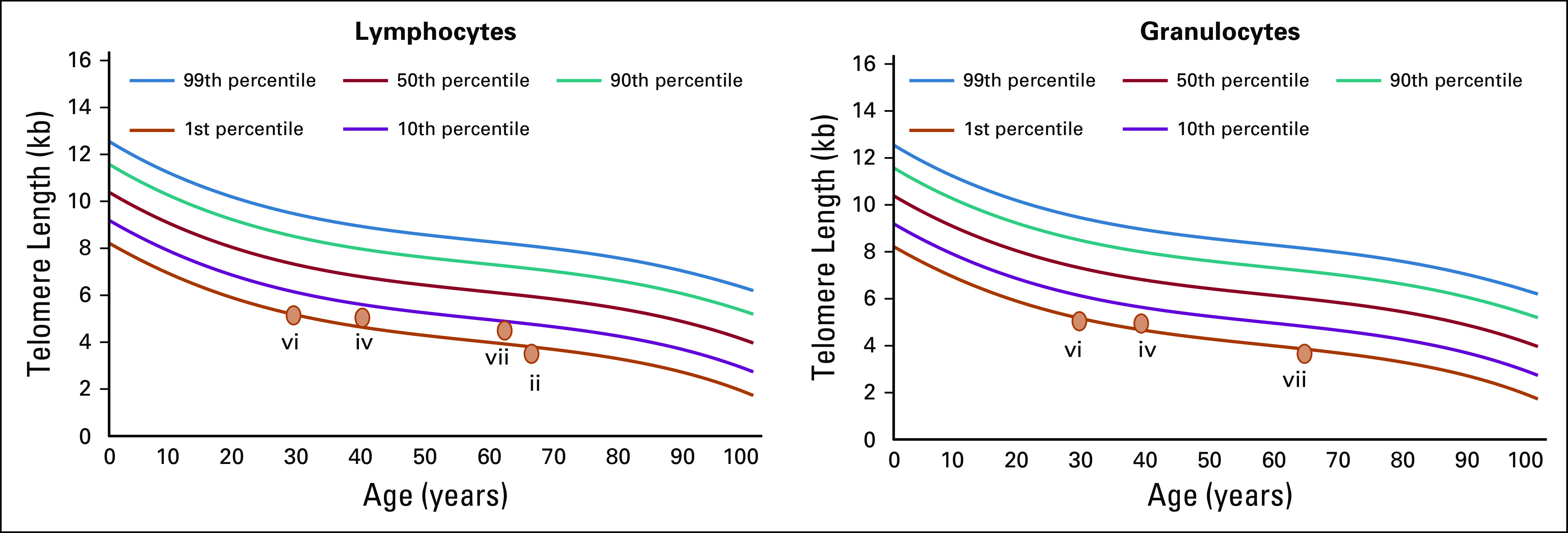
Present cytometric fluorescence in situ hybridization lymphocyte results for individuals ii, iv, vi, and vii and granulocyte results for individuals iv, vi, and vii.

**FIG 2.**
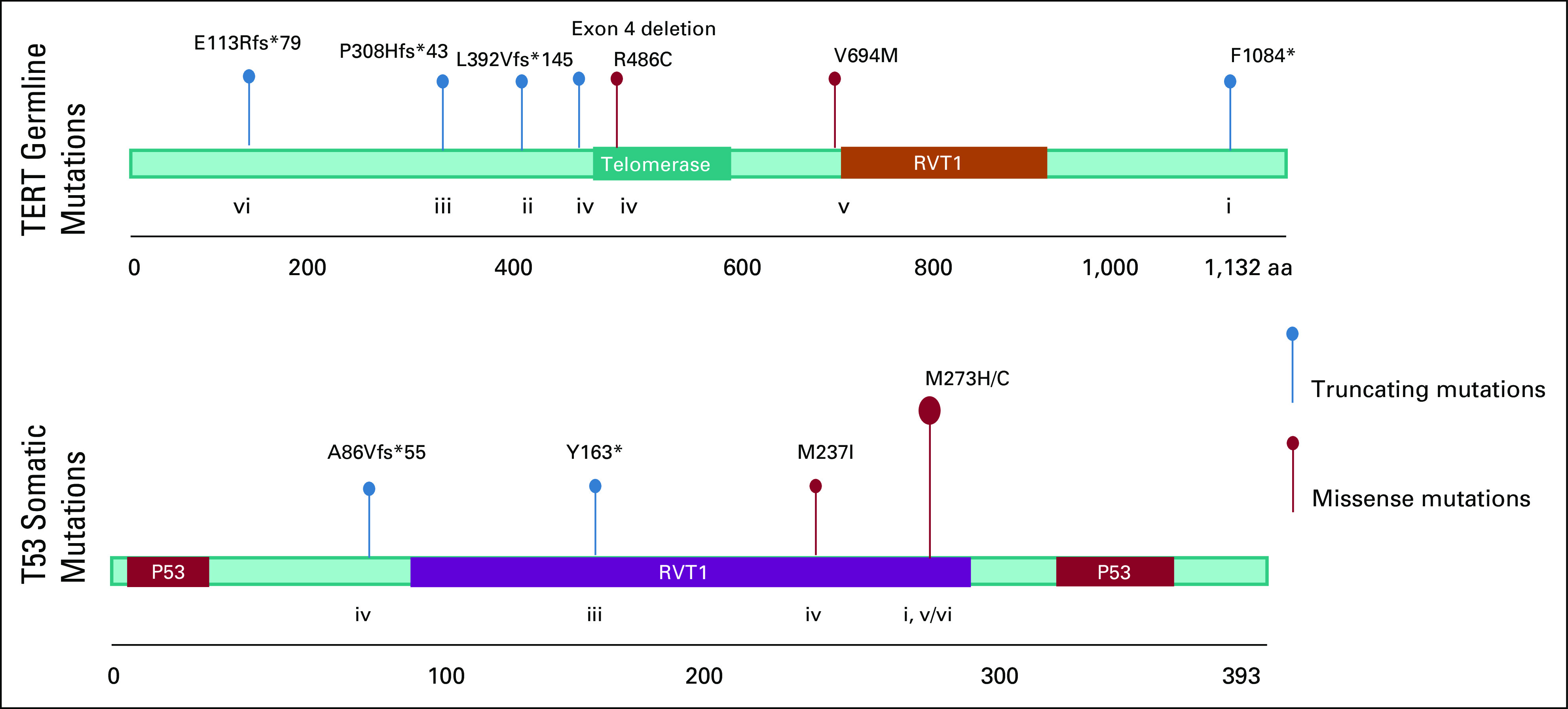
Germline *TERT* mutational lollipop illustration (n = 7) and somatic *TP53* mutational lollipop illustration (n = 5). Individuals ii and vii were without tumor specimens available for sequencing, and individual vi was found to have two somatic *TP53* mutations. aa, amino acid.

For two individuals, telomere length testing through cytometric fluorescence in situ hybridization identified telomere lengths in the 1st percentile or less; in two additional individuals, telomere lengths were in the 10th percentile or less; and in one additional individual, telomere length testing failed because of low blood counts that did not recover after chemotherapy (Fig 1). For two individuals, it was not possible to obtain telomere lengths. Somatic mutation analysis of all five individuals with germline *TERT* mutations and tumor specimens available for analysis showed somatic *TP53* driver mutations, which are generally associated with poor prognosis^28-30^ (Fig 2).

## DISCUSSION

An understanding of the genetic basis that contributes to therapeutic sensitivities is important for oncology patients who receive chemotherapy, radiation, and other biologic therapeutics. The identification of patients with known therapeutic vulnerabilities is important for patient care. Patients with telomere syndromes may manifest overt or subtle clinical findings.^5^ Moreover, for patients with both obvious and subtle telomere syndromes, exquisite treatment sensitivities have been reported.^6,7^ Although only one in 1,571 individuals in this series of patients with advanced cancer showed a germline *TERT* mutation, this could translate to more than 1,000 patients diagnosed with malignancies a year in the United States who may have increased therapeutic sensitivities. Because telomere syndrome disorders are complex and exhibit anticipation, incomplete penetrance, multiple genes that underpin disease, recognized modifiers, and both autosomal dominant and autosomal recessive patterns of inheritance, this estimate will be refined with time. However, until a clearer picture is possible, it seems reasonable for individuals with constitutional germline *TERT* mutations to be considered for monitoring given the potential long-term sequalae from chemotherapy and radiation as well as increased organ-specific damage.^7,8,31^

Integration of germline, somatic, and clinical data for patients in our cohort was also notable for somatic *TP53* mutations and a younger age at cancer diagnosis compared with the general population. Moreover, multiple *TERT* single nucleotide polymorphisms have been shown to be associated with telomere length and breast and ovarian cancer risk.^31^ All this information together with telomere lengths may provide insights for stratifying patients with regard to age at presentation, outcome, and tumor evolution.

Future studies that interrogate for germline mutations in other genes implicated in telomere biology (ie, *CTC1*, *DKC1*, *NHP2*, *NOP10*, *TERC*, *TINF2*, *RTEL1*) in the setting of cancer may reveal additional individuals with potential therapeutic sensitivities. In addition, studies that attempt to elucidate a clearer role in oncogenesis are needed. In the context of Li Fraumeni syndrome (ie, individuals with germline *TP53* mutations), short telomeres are associated with earlier onset of cancer, which likely results from genomic instability.^32^ Somatic data from this study also supports the interplay between *TP53* and telomere regulatory genes.
